# Green Synthesis of Silver Nanoparticles using *Achillea biebersteinii* Flower Extract and Its Anti-Angiogenic Properties in the Rat Aortic Ring Model

**DOI:** 10.3390/molecules19044624

**Published:** 2014-04-15

**Authors:** Javad Baharara, Farideh Namvar, Tayebe Ramezani, Nasrin Hosseini, Rosfarizan Mohamad

**Affiliations:** 1 Research Center for Animal Development Applied Biology, Mashhad Branch, Islamic Azad University, Mashhad, Iran; E-Mails: baharara@yahoo.com (J.B.); ninahosseini75@yahoo.com (N.H.); 2 Department of Biology, Mashhad Branch, Islamic Azad University, Mashhad, Iran; 3 Institute of Tropical Forestry and Forest Products (INTROP), Universiti Putra Malaysia, Serdang, Selangor 43400 UPM, Malaysia; E-Mail: farizanmohd@gmail.com; 4 Research Center for Animal Development Applied Biology & Department of Medicine, Mashhad Branch, Islamic Azad University, Mashhad, Iran; 5 Faculty of Biological Sciences, Kharazmi University, Tehran 31979-37551, Iran; E-Mail: tayeberamezani@gmail.com; 6 Department of Bioprocess Technology, Faculty of Biotechnology and Biomolecular Sciences, Universiti Putra Malaysia, Serdang, Selangor 43400 UPM, Malaysia

**Keywords:** *Achillea biebersteinii*, angiogenesis, green synthesis, silver nanoparticle

## Abstract

Silver nanoparticles display unique physical and biological properties which have attracted intensive research interest because of their important medical applications. In this study silver nanoparticles (*Ab*.Ag-NPs) were synthesized for biomedical applications using a completely green biosynthetic method using *Achillea biebersteinii* flowers extract. The structure and properties of *Ab*.Ag-NPs were investigated using UV-visible spectroscopic techniques, transmission electron microscopy (TEM), zeta potential and energy dispersive X-ray spectrometers (EDS). The UV-visible spectroscopic analysis showed the absorbance peak at 460 nm, which indicates the synthesis of silver nanoparticles. The average particle diameter as determined by TEM was found to be 12 ± 2 nm. The zeta potential analysis indicated that *Ab*.Ag-NPs have good stability EDX analysis also exhibits presentation of silver element. As angiogenesis is an important phenomenon and as growth factors imbalance in this process causes the acceleration of several diseases including cancer, the anti-angiogenic properties of *Ab*.Ag-NPs were evaluated using the rat aortic ring model. The results showed that *Ab*.Ag-NPs (200 μg/mL) lead to a 50% reduction in the length and number of vessel-like structures. The synthesized silver nanoparticles from the *Achillea biebersteinii* flowers extract, which do not involve any harmful chemicals were well-dispersed and stabilized through this green method and showed potential therapeutic benefits against angiogenesis.

## 1. Introduction

Currently, improving and protecting our environment using green chemistry have become important issues in many fields of research [1]. In the field of nano-science, the use of various biological units instead of toxic chemicals for the reduction and stabilization of metal nanoparticles, has received extensive attention [2]. Biological entities, such as bacteria [3], fungi [4], yeasts [5], algae [6] or plants [7,8], have been reported as serving as both reducing and stabilizing agents. Among these possible bio- resources, biologically active products from plant resources represent excellent scaffolds for this purpose [7]. Plant extracts, which are rich in bioactive compounds, have recently been used for NPS green synthesis. Many different plant leaves and herbs for the synthesis of nanoparticles have been reported [9]. The mechanism of biosynthesis of nanoparticles in plants may be associated with the phytoremediation concept in plants [7]. The genus *Achillea* (Asteraceae) comprising ~85 species has some interesting properties and has been known to be ethnopharmacologically used in folk medicine for various purposes such as a diuretic, for abdominal pain, against diarrhea, flatulence, as an emmenagogue and for wound healing [10]. Several biological activity studies have been performed on various *Achillea* species, including antibacterial, antioxidant, anti-inflammatory and antispasmodic activities [11,12]. Saeidnia *et al.* reviewed the medicinal properties of various species of *Achillea* [13], *while* Si *et al.* reviewed the structures and biological properties of the known phytochemical constituents of the *Achillea* species [14].

The most promising approach for generating new fields in biomedical sciences is the pharmaceutical application of NPs [1]. Among metal NPs, nanosilver exhibits outstanding physical, chemical and biological properties [15]. Ag-NPs have potential in treating a variety of diseases, including retinal neovascularization, immunodeficiency syndrome [16], infection [17] and cancer [18]. The growth factors imbalance is involved in the acceleration of several diseases including cancer, ocular, and inflammatory diseases [19]. A promising methodology to hinder the progression of these diseases can be through inhibiting angiogenesis.

The present study describes a green method using flowers of *Achillea biebersteinii* extracts for the biosynthesis of Ag-NPs without any additive protecting nanoparticles from aggregating, template shaping nanoparticles or accelerants. The current simple biosynthetic method using precursors from flowers of *Achillea biebersteinii* provides high-yield nanosized materials. The characterization and formation mechanisms of Ag-NPs are discussed; moreover, the effects of synthesized NPs in the capillary like formation of the rat aortic ring model were evaluated by observing the morphology.

## 2. Results and Discussion

### 2.1. Green Synthesis of Ag-NPs and Characterization

**Figure 1:**
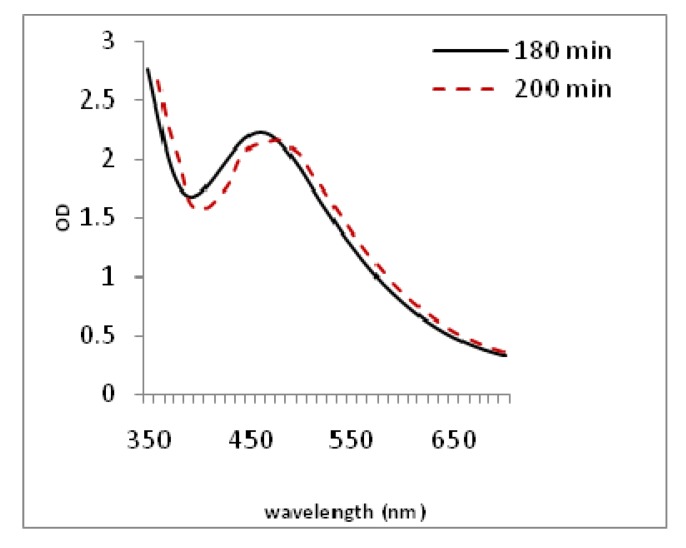
UV-visible absorption spectra of *Ab*.Ag-NPs in different times.

During the addition of the plant extract to the aqueous AgNO_3_ solution, the color of the solution reaction slowly changed from yellow-wish to a dark brown color, indicating the formation of Ag-NPs. The appearance of the dark brown color may be due to the excitation of the surface plasmon resonance (SPR) effect and the reduction of AgNO_3_ [20].The UV-Vis absorption spectrum showed a strong absorption peak centered at 460 nm which indicated the formation of Ag-NPs. (Figure 1). This absorption is close to the absorbance peaks reported by Jha *et al.* [21] and Arunachalam *et al.* [22] to synthesize silver nanoparticle using green methods. These nanoparticles were well dispersed without adding different physical and chemical capping agents. The synthesized Ag-NPs from the flowers of *Achillea biebersteinii* extracts (*Ab*.Ag-NPs) were observed to be very stable in the solution, even one month after their synthesis. Overall the best synthesized nanoparticles were mixed in a ratio of 1 plant extract to 10 AgNO_3_ (5 mM) in 180 min.

### 2.2. Morphology and Size Distribution of Nanoparticles

**Figure 2:**
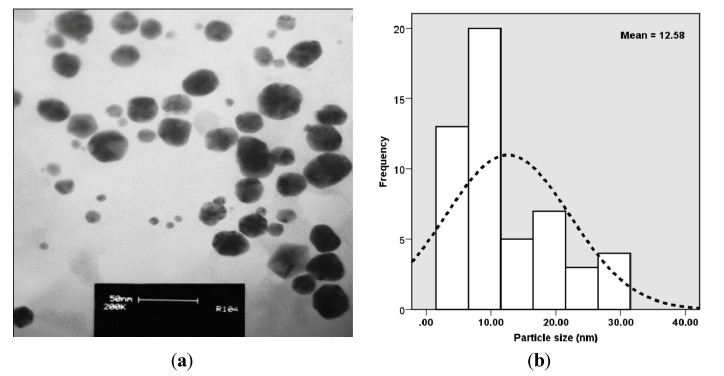
(**a**) TEM micrograph; and (**b**) particle size distributions of biosynthesized *Ab*.Ag-NPs.

The TEM image Figure 2a and particle size distribution graph Figure 2b show that the Ag-NPs formed were well dispersed with different shapes such as hexagonal, pentagonal and spherical structures with particle sizes ranging from 5 to 35 nm with a mean size of 12.58 nm. The presence of secondary materials can be seen from the capping with dark shades on the surface of nanoparticles, which may be assigned to the bio-compounds present in the Ab extract. The bio-components within the Ab not only result in the efficient reduction of silver salts to nanoparticles, but, likewise as an appropriate capping agent, inhibiting them from aggregation [23]. The different bio-compounds present in Ab extract such as polysaccharides, polyphenols, and proteins can produce nanoparticles with different shapes [24].

**Figure 3:**
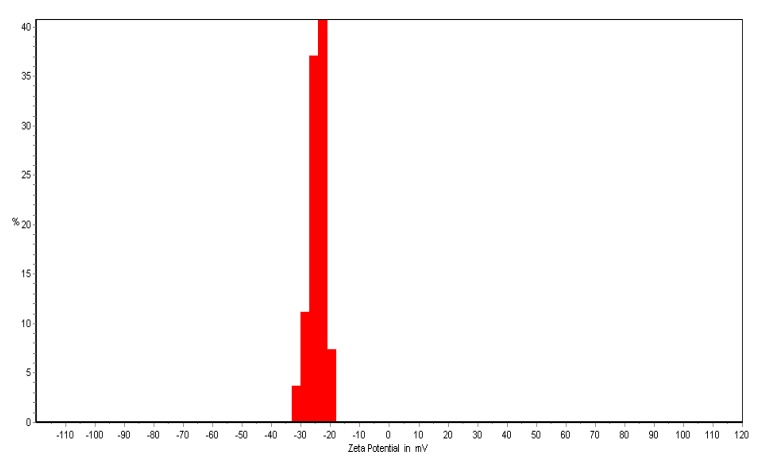
The zeta potential of the biosynthesized *Ab*.Ag-NPs.

The zeta potential test was used to measure the electrophoretic mobility of each nanoparticle sample. Complex zeta potential is a parameter that is used in the study of the surface charges and stability of NPs. These charges greatly influence on the particle distribution and cellular uptake. A high absolute zeta potential value indicates a high electric charge on the surface of the NPs. It describes strong repellent forces among the particles, which prevent aggregation and lead to stabilizing the NPs in the medium. The zeta potential of the nanoparticles formulated was only measured in systems that did not sediment after overnight equilibration. At natural provision (pH = 7.2), the values for the zeta potential were −20 to −40 Mv. This result revaluated the synthesized silver nanoparticle that were stable due to the electrostatic repulsion without adding a different physical or chemical capping agent. This is very important for use for therapeutic propose (Figure 3).

**Figure 4:**
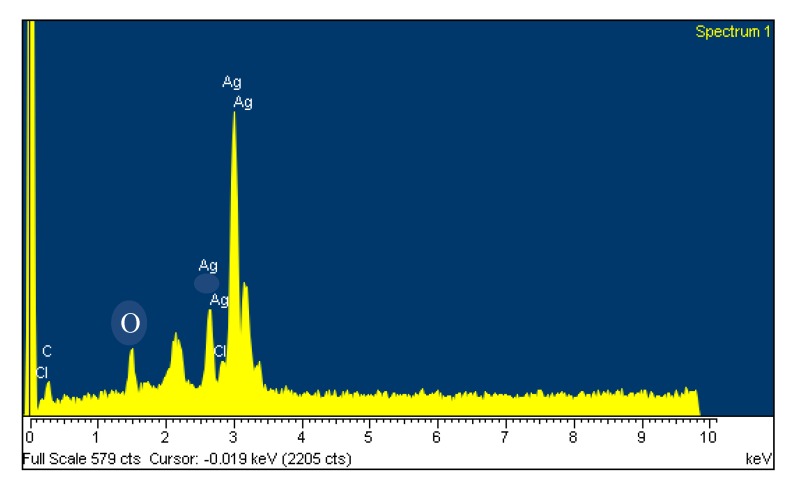
EDS spectrum of bi-synthesized *Ab*.Ag-NPs.

The EDS spectrum detailed from the *Ab*.Ag-NPs is shown in Figure 4. The EDS spectrum reveals a strong signal in the silver region and confirms the formation of silver nanoparticles. The Ag-NPs generally show a sharp optical absorption peak approximately at approximately 3 KeV due to the surface plasmon resonance. The other signals below 3 KeV are related to the binding energies of bio-compounds on the surface of the Ag-NPs. Therefore, the EDS spectrum of *Ab*.Ag-NPs confirms the presence of elemental compounds of the *Ab* extract and Ag nanoparticles without any impurity peaks. The results indicate that the synthesized the *Ab*.Ag-NPs are of high purity.

### 2.3. Effects of Ab.Ag-NPs on Number and Length of Capillary like Structures in Aortic Ring Assay

**Figure 5:**
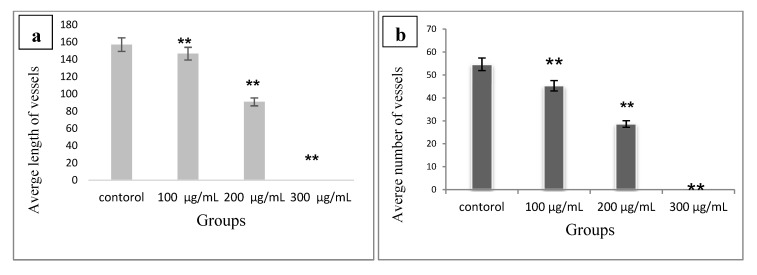
The effect of different concentrations of silver nanoparticles on length (**a**) and number (**b**) of small vessels in the aortic ring model (** *p* < 0.001).

According to the results, high doses of *Ab*.Ag-NPs (200 μg/mL) lead to destroy the capillary like and cell necrosis in the first hours of treatment a low dose (below 100 μg/mL) did not show any effects on the length and number of the vessels formed. The *Ab*.Ag-NPs (200 μg/mL) lead to about a 50 percent decrease in number and length of the vessels (Figure 5a,b).

### 2.4. Effects of the Ab.Ag-NPs on Vessel’s Morphology

**Figure 6:**
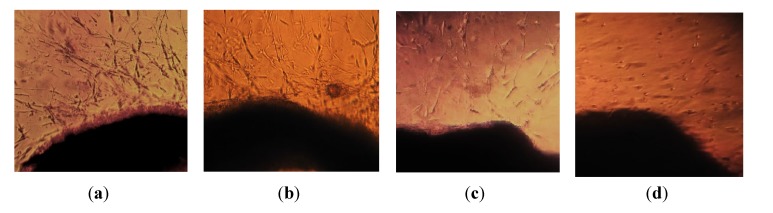
The inhibitory effects of silver nanoparticles on appearances vascular like appendages from the aortic rings. (**a**) Control; (**b**) The concentration of 50 μg/mL wasn’t able to inhibit destroy vessel; (**c**) modular inhibitory effect on 200 μg/mL; (**d**) Strong inhibitory effect 300 μg/mL.

Microscopic observations of treated vessels showed distinct morphological changes indicating an unhealthy organization in 200 μg/mL and higher concentrations, whereas the control appeared normally (Figure 6a,c). After treatment with toxic dosages of nanoparticles, the endothelial cells that make a vessel like structure, appeared to be clustered with a few cellular extensions, and cell-spreading patterns were restricted compared to the control cells, this could be due to disturbances in the cytoskeletal functions as a consequence of nanoparticle treatment. Similar results were observed by other groups. Low doses of *Ab*.Ag-NPs did not show any effect on the vascular organization (Figure 6b). The evidence showed that angiogenesis is critical for the growth and progression of solid tumors because growth in tumor mass beyond 2 to 3 mm is often caused by an increase in the formation of new blood vessels which is probably essential for the delivery of nutrients and oxygen to the tumor microenvironment [25]. Therefore, anti-angiogenic therapy represents one of the most promising approaches to control tumor growth and invasiveness. Different *in vivo* and *in vitro* assays have been used so far in order to recognize the screen angiogenic activators and inhibitors and for the development of the vascular system [26]. Commonly used *in vivo* models of angiogenesis include the chorioallantoïc membrane of the chick embryo (CAM assay), rabbit cornea, aortic ring and the matrigel implant assay [27]. Nicosia and Ottinetti confirmed that rat aorta rings reproducibly generate microvessel outgrowths in fibrin or collagen gels, and offer a sensitive assay for the investigation of angiogenic agonists and antagonists in a chemically defined environment. Thus, this system bridges the gap between *in vitro* and *in vivo* [27]. Angiogenic sprouting in aortic cultures is followed by migration of adventitial macrophages and fibroblasts into the periaortic gel matrix. Once vessel sprouts occur from the explants, they generate networks by elongating, forming anastomotic loops and branching through endothelial migration and proliferation [27]. The angiogenic or anti-angiogenic properties of the agent can be further demonstrated by embedding aortic endothelial cells in collagen gel [28]. The present study demonstrated that silver nanoparticles have cytotoxic effects on the endothelial cells. The *Ab*.Ag-NPs showed dose-dependent cytotoxicity against the endothelial cells. Another study also showed that Ag-NPs lead to subtle obstructive effects to the microcirculation in the chick embryo chorioallantoic membrane (CAM). These effects occurred without loss of embryo viability and were associated with the partial preservation of the capillary diameters and connectivity [29]. Ag-NPs could inhibit the vascular endothelial growth factor (VEGF)—induced bovine retinal endothelial cells like migration, proliferation and capillary-like tube formation. Moreover, the Ag-NPs effectively inhibited the formation of new blood micro vessels induced by VEGF in the mouse Matrigel plug assay [19,30]. Similar studies have confirmed the inhibitory effect of Ag-NPs on the vascular permeability induced by VEGF, interleukin (IL)-1β, in retinal endothelial cells [30]. These reports were consistent with our finding. The mechanism may be due to induced apoptosis that affects the proteins and enzymes with thiol groups like thioredoxin, SOD, thioredoxin peroxidase and glutathione, which are responsible for neutralizing the oxidative stress of Reactive Oxygen Species (ROS) that are largely generated by mitochondrial energy metabolism [18].

## 3. Experimental

### 3.1. Materials

AgNO_3_ was purchased from Merck (Darmstadt, Germany). Specimens of *Achillea biebersteinii* were obtained from a local source. All the solutions were prepared with double distilled water. Other chemicals were of analytical grade.

### 3.2. Extraction Preparation

The identification of *Achillea biebersteinii* was confirmed by a plant taxonomist from the herbarium division of Ferdowsi University of Mashhad, where a sample was deposited with the voucher specimen number 34,516. The flower parts of freshl *Achillea biebersteinii* were washed thoroughly three times with double distilled water, and air-dried in the shade at room temperature for 14 days. For the production of the extract, ground, freeze-dried *Achillea biebersteinii* samples (about 1 g) were boiled with DDW (100 mL) in an Erlenmeyer flask while being continuously stirred for 15 min. The extract was cooled to room temperature, filtered and used for the synthesis of Ag-NPs.

### 3.3. Synthesis and Characterization of Ag-NPs

#### 3.3.1. The Biosynthesis of Silver Nanoparticles (Ag-NPs)

For the preparation of silver nanoparticles, AgNO_3_ aqueous solution (5 mM) was mixed with different volume of plant extract under continuous stirring at 40 °C. The color of the solution slowly changed from grayish yellow to dark brown indicating the formation of silver nanoparticles.

#### 3.3.2. Characterization Methods

The prepared Ag-NPs were monitored by UV-vis spectroscopy using a UV-vis spectrophotometer system (Epoch Biotech, Winooski, VT, USA) in the wavelength range from 300–700 nm. Energy Dispersive X-ray spectroscopy (EDS) was performed (XL 30; Philips, Eindhoven, The Netherlands) to study the composition of the product. The morphology and size of the synthesized Ag-NPs were measured using a field emission scanning electron microscope by placing a drop of the nanoparticles solution on the carbon coated copper gird and drying in air before transferring to the microscope. The size of the distribution and the average size of 50 nanoparticles were estimated on the basis of three TEM images with the assistance of Sigma-Scan Pro software (SPSS IBM, Statistics 20, IBM Corporation, Endicott, NY, USA) The zeta-potentials of Ag-NPs in water were evaluated using CAD (Zeta compact, Les Essarts-le-Roi, France). Samples were sonicated for 5 min before measurement to ensure that the particles were well dispersed and that the dispersion was homogeneous.

### 3.4. Aortic Ring Assay

#### 3.4.1. Type I Collagen Extraction

Type I collagen was extracted from rat-tails. The rat tails were obtained from the Research Center for Animal Development Applied Biology, Islamic Azad University of Mashhad. Type I collagen was first extracted using the modified method by Techatanawat *et al.* [31]. Briefly, the skin was removed from the rat tail and the tendon taken from the tail was transferred to a sterile bottle with a stir bar and dissolved by adding about 75 mL of distilled water (pH = 4) and stirred for 2 days at 4 °C. Then the resulting viscous solution was centrifuged and stored at 4 °C for at most three months. The total protein concentrations of the samples were determined using the Bradford protein assay.

#### 3.4.2. Preparation of Collagen Matrix and Aortic Ring Culture

Angiogenesis was investigated by culturing the aortic ring in three-dimensional matrix gels. The aortas were removed from 2–8 weeks old rats and immediately moved to a culture dish containing cold PBS. The periaortic fibroadipose tissue was removed. The aorta was sectioned in 1–2 mm-long rings and was embedded in a gel of rat tail collagen. The final collagen matrix was obtained by mixing 8 volumes of collagen (4 mg/mL) with 1 volume of 10× DMEM, and 1 volume of NaHCO_3_, and kept at 37 °C for 20 min. Then, 50 µL of DMEM containing 30% FBS, and 100 U/mL penicillin-streptomycin was added to each well. The cultures were kept at 37 °C in a humidified environment for a week and examined every second day with an Inverted Microscope (Biomed, Seoul, Korea) at an appropriate magnification.

#### 3.4.3. Anti-Angiogenesis Effects of Ag-NPs

Three days after the aortic ring culture when the first small vessels like protrusions became visible silver nanoparticles were added to the wells with concentrations of 50, 100, 200 and 300 μg/mL, also some wells were considered as well as the control. Over the 24 h the results were photographed using an inverse microscope and digital camera (Canon, Tokyo, Japan). The length and number of the small vessels like protrusions before and 24 h after treatment were obtained by image analysis performed with the ImageJ software package24. Statistical analyses were performed using SPSS software. The results were shown as mean ± SD and *p* < 0.05 was accepted as the level of significance.

## 4. Conclusions

Angiogenesis has a central role in myocardial infarction, atherosclerosis, carcinogenesis, limb and cardiac ischemia, and tumor growth. The development of new therapeutic strategies aimed at reducing angiogenesis could therefore have serious clinical implications. In the present study Ag-NPs with an average size of 12 ± 2 nm were synthesized by means of a green method using an aqueous extract containing phytochemicals as the reducing agent and efficient stabilizer. The characteristics of the obtained *Ab*.Ag-NPs were studied using, UV-visible, EDX, TEM and zeta potential techniques. The biosynthesis of Ag-NPs using green resources is a simple, environmentally friendly, low-cost and non-toxic approach. The *Ab*.Ag-NPs showed anti-angiogenic properties in the rat aortic ring model. Our findings indicate that Ag-NP may have potential therapeutic benefits as anti-angiogenesis agents. Therefore these green Ag-NPs may potentially provide an attractive and cheap therapeutic alternative for treating various conditions characterized by abnormal angiogenesis.
